# Collection of Fees

**Published:** 1900-10-15

**Authors:** 


### COLLECTION OF FEES.

One of our young friends told us recently, with some pardonable pride, that as soon as he opened his office in a city in the Province of Quebec, where he was a perfect stranger, and without resorting to any of the advertiser's tricks of trade, he quickly acquired a large practice. It is an experience which many of us have "enjoyed," and which elated our souls beyond measure, until its hollowness later on was revealed. Our young friend had a little capital in the bank, which he invested in neat furnishings, etc. His personal manners are beyond reproach, his equipments first-class. He soon found he had among his patients a large number of that class who live chiefly upon their own wits and the innocence of their creditors, and who constitute a certain *creme de la creme* of fashionable society. After four years he began to waken to the fact that he was having more struggle to get his money than he had to get his practice; that for every dollar in his pocket he had fifty on his books, and to day he is really worse off than when he began, so far as his cash revenue is concerned. In fact, he is barely able to make both ends meet. But, glorious thought! he has over four thousand dollars on his books—and there they will likely remain! In starting life with honest purpose, our young friend overlooked the important fact that while he put his conscience into his daily labors, he failed to put some of his zeal into his collections. He failed to remember that a beginner is pretty certain to attract the impecunious and the dead beat, so many of whom pose as members of the social set of our cities. His chief anxiety was to show his patients how well he would do his work—a very noble ambition. But as we told him, he reminded us of our own and others' earliest experiences, as well as of Mme. Cibot, the housekeeper in one of Balzac's novels, who set a thousand times as much store on being appreciated at her true value as she did upon being paid.—*Editorial, in Dominion Dental Journal.*

---

Painless Operations.—When you do not wish to use cocain solutions or other drugs that are liable to be poisonous, take a pair of pointed pliers and dip in a solution of chloral-camphor and pass it gently around the root of the tooth(freed from blood and saliva), and an operation that is usually very painful will in many cases be entirely painless, in others almost so, and you have no bad-smelling drug in the mouth.—*A. W. Harlan, Den. Review.*

